# The Hidden Burden of Gastroparesis in Chronic Kidney Disease: Evidence from Inpatient and Outpatient Cohorts for Personalized Care

**DOI:** 10.3390/jpm15120600

**Published:** 2025-12-04

**Authors:** Xiaoliang Wang, Omar Almetwali, Armando Marino-Melendez, Darwin Tan, Jiayan Wang, Gengqing Song

**Affiliations:** 1 Digestive Disease & Surgery Institute, Cleveland Clinic, Cleveland, OH 44195, USA; wangx12@ccf.org (X.W.); marinoa4@ccf.org (A.M.-M.); 2 Internal Medicine, Joan C. Edwards School of Medicine, Marshall University, Huntington, WV 25701, USA; almetwaliot@upmc.edu; 3 Department of Internal Medicine, University of Pittsburgh Physicians, Pittsburgh, PA 15213, USA; 4 Case Western Reserve University, Cleveland, OH 44106, USA; dxt298@case.edu; 5 Division of Nephrology & Hypertension, Case Western Reserve University and University Hospital Cleveland Medical Center, Cleveland, OH 44106, USA; jiayan.wang@uhhospitals.org; 6 Department of Gastroenterology and Hepatology, Metrohealth Medical Center, Case Western Reserve University, Cleveland, OH 44109, USA

**Keywords:** chronic kidney disease, gastroparesis, inpatient, outpatient, risk factors

## Abstract

**Background/Objectives:** Patients with chronic kidney disease (CKD) frequently experience upper gastrointestinal (GI) symptoms such as epigastric discomfort, nausea, vomiting, and early satiety. These symptoms can contribute to malabsorption and intermittent dehydration, ultimately accelerating the decline of residual renal function. However, they are often attributed to electrolyte imbalances or fluid overload, and the possibility of underlying gastroparesis is frequently overlooked by both patients and caregivers. This study aimed to provide new insights into the relationship between CKD and gastroparesis through a dual, population-based retrospective analysis that incorporated both inpatient and outpatient data. **Methods:** From the National Inpatient Sample (NIS) database, 3,579,372 patients diagnosed with gastroparesis, with or without CKD, were identified. From the TriNetX database, 6,263,251 patients presenting to ambulatory clinics with a chief complaint of nausea and vomiting were included. In both datasets, gastroparesis was defined using ICD-10-CM codes. **Results:** In the inpatient cohort, the prevalence of gastroparesis increased in proportion to CKD severity, with the highest likelihood observed in advanced stages compared to patients without CKD. An increased risk of gastroparesis was also observed in the outpatient CKD cohort from an independent TriNetX database, while the severity-dependent phenotype was not consistent. However, after rigorous propensity score matching, advanced CKD remained significantly associated with higher odds of gastroparesis, with the greatest risk observed in patients with end-stage renal disease (ESRD). **Conclusions:** These findings, validated across two large and independent datasets representing both inpatient and outpatient populations, demonstrate a consistent association between CKD severity and gastroparesis. They highlight the importance of routine screening and early management of gastroparesis in patients with advanced CKD to improve outcomes and reduce disease burden for CKD patients with sign of early satiety or dyspepsia.

## 1. Introduction

According to a 2023 CDC report, CKD affects approximately 14% of adults in the United States—over 35 million individuals—with the majority of cases occurring in those over the age of 65 [1]. Patients with CKD, particularly in the later stages, frequently report upper GI symptoms such as nausea, early satiety, bloating, and abdominal discomfort. These symptoms are often exacerbated during episodes of fluid imbalance or dehydration, potentially leading to further renal injury and accelerating loss of residual kidney function. However, such symptoms are frequently attributed to renal insufficiency-related electrolyte and fluid disturbances and are therefore overlooked by both patients and caregivers. As a result, only individuals with severe or persistent symptoms are typically referred to gastroenterology for further evaluation [2].

Survey data from 2015–2018 estimated that the national prevalence of CKD in U.S. adults is at 14.9%. Within this population, up to 70% of patients reported symptoms suggestive of gastric dysmotility, including nausea, vomiting, early satiety, and epigastric pain [2]. Several mechanisms have been proposed to explain these associations, including comorbid conditions such as gastritis, peptic ulcer disease (PUD), and asymptomatic gastric polyps [3,4]. Few studies have documented the potential association between renal disease and gastroparesis. One literature review reported the high prevalence of dysmotility-like gastrointestinal symptoms and delayed gastric emptying among patients with CKD [5]. In addition, two separate studies have described a similarly high prevalence of gastroparesis in patients with ESRD. While these studies have suggested that patients with CKD may be at increased risk of developing gastroparesis, the evidence has been inconclusive, as observed delayed gastric emptying has not always correlated with either CKD severity or the frequency of reported GI complaints [5,6,7].

Gastroparesis is a clinical condition that affects the normal functioning of the stomach by delaying gastric emptying without evidence of mechanical obstruction, resulting in impaired digestion and nutrient absorption [8]. The etiology can be broadly subclassified as diabetic [9] versus non-diabetic, with the latter encompassing both idiopathic causes, which is attributed with the highest disease burden [10], as well as systemic disease, such as Connective tissue disease (Scleroderma), Neuromuscular (Parkinsonism), Vascular disorders (Mesenteric ischemia), and pharmacological side effects to name a few [11]. Diabetes has long been recognized as a leading cause of gastroparesis [12]. The prevalence of gastroparesis among patients with diabetes ranges from 1% to 5.2%, depending on the type of diabetes, compared with approximately 0.2% in the general population [12,13]. Moreover, clinical symptoms consistent with gastroparesis, as assessed by the Gastroparesis Cardinal Symptom Index (GCSI), are reported in more than 10.8% of diabetic patients [14]. Long-term diabetes mellitus is a major risk factor for gastroparesis and is also the leading cause of CKD, commonly referred to as diabetic kidney disease. Even among diabetic patients receiving appropriate treatment, the risk of progression to end-stage renal disease (ESRD) remains significantly higher than in non-diabetic individuals [15,16]. The underlying mechanisms are thought to involve glomerular hyperfiltration, chronic inflammation, and oxidative stress–induced fibrosis affecting both glomeruli and renal tubules [17]. It has been reported that more than 30% of diabetic patients develop proteinuria after 25 years of diabetes, and among these, approximately 31% progress to ESRD if left untreated [18]. In 2015, the annual incidence of ESRD among diabetic patients exceeded 1000 cases per million population [18].

This disorder manifests through a variety of non-specific symptoms, including persistent nausea, vomiting, bloating, and early satiety, making it challenging for affected individuals to maintain a healthy diet and further impacting their quality of life [19]. However, despite a well-established consequential relationship between some of the most common etiologies, such as diabetes [8,20], and gastroparesis, the over-reliance on symptomatic clues of underlying gastric motility defect is not always a straightforward process, and at times, paradoxical [21]. The diagnosis of gastroparesis in suspected patients should begin with the exclusion of mechanical obstruction through imaging studies or esophagogastroduodenoscopy (EGD) [8]. Once obstruction is ruled out, a gastric emptying scintigraphy is performed to confirm delayed gastric emptying and assess its severity. Delayed gastric emptying is defined as greater than 10% gastric retention at 4 h after ingestion of a 255 kcal, 2% fat egg substitute meal, or greater than 60% retention at 2 h when using a standard low-fat scrambled egg meal [22]. Although patients with diabetes have a significantly higher risk of developing gastroparesis, routine screening using gastric emptying tests is not recommended; testing should be reserved for patients presenting with postprandial symptoms such as nausea, vomiting, abdominal pain, bloating, or early satiety [8].

A 2020 study estimated that the prevalence of gastroparesis in the US was 0.16% between 1999–2014 [23]; this figure is likely an underestimation of the true disease burden due to misdiagnosis as potentially coexisting conditions such as functional dyspepsia (FD), Eosinophilic esophagitis (EE), and Gastroesophageal Reflux Disease (GERD) [24,25,26,27], while only a fraction of those patients exhibiting these symptoms undergo diagnostic testing [28]. Moreover, delayed diagnosis can be attributed to a subclinical disease course and a protracted onset of symptoms indicative of gastroparesis. Patients with type 1 diabetes mellitus were found to have a higher incidence of gastroparesis compared to their counterparts diagnosed with type 2 diabetes mellitus, However, patients with type 2 diabetes mellitus exhibited more pronounced symptoms and suffered extensive microvascular complications, such as neuropathy and retinopathy [8]. The diagnostic process entails a comprehensive evaluation of symptoms, medical history, luminal evaluation, and functional testing through gastric emptying studies. Despite the increasing prevalence and significant disease burden [29], the underlying mechanisms of gastroparesis are not yet fully understood, thus limiting therapeutic options and further delaying adequate care.

Due to the non-specific symptoms of gastroparesis and the limited, often unsatisfactory treatment options currently available, early diagnosis and identification of risk factors are crucial for enabling timely interventions and improving patient outcomes. This is particularly important for individuals with CKD, especially those in the early stages who may present with only mild symptoms of gastroparesis. Early recognition and management in this population could help reduce the incidence of recurrent dehydration–related renal injury, prevent malnutrition, and potentially slow CKD progression.

Despite these implications, the relationship between CKD and gastroparesis requires further exploration through large, retrospective clinical studies. However, because confirmed diagnoses of gastroparesis remain relatively uncommon among patients with CKD, such studies would require a large sample size and substantial time investment. Therefore, utilizing national patient databases represents an efficient and feasible initial approach to investigate this association. Our study aims to evaluate whether CKD is associated with a higher prevalence of gastroparesis by leveraging two large databases that capture both inpatient and outpatient populations across the United States.

## 2. Materials and Methods

### 2.1. Database

**For the inpatient cohort**, a retrospective analysis was performed using the National Inpatient Sample (NIS) database developed by the Healthcare Cost and Utilization Project (HCUP). NIS is the largest publicly available all-payer inpatient healthcare database designed to estimate inpatient utilization, access, cost, quality, and outcomes, with unweighted data from around 7 million hospital stays each year. The NIS approximates a 20% stratified sample of all discharges from US community hospitals, excluding rehabilitation and long-term acute care hospitals.

**For the outpatient cohort**, a retrospective analysis was performed using the TriNetX (TriNetX LLC, Cambridge, MA, USA) database, a global health research network that provides real-time de-identified electronic health record (EHR) data from various collaborating healthcare organizations (Within the US). This database provides access to longitudinal clinical data that spans outpatient settings, including outpatient medication, lab results, and the ability to evaluate for disease progression, providing a unique and more comprehensive assessment timeline.

Both the NIS and TriNetX databases are fully de-identified datasets; therefore, Institutional Review Board approval was not required for this study. All participating institutions have authorized the use of these databases for research purposes. In addition, all investigators involved in data collection and analysis completed the required NIS data use and data safety training and were certified in proper data handling procedures.

### 2.2. Data Collection and Outcomes

For the inpatients database, adult inpatient records were included in this study using the NIS database. Patients diagnosed with gastroparesis (ICD-10-CM K3184) with or without CKD (ICD-10-CM N18.1-9) were compared to patients without gastroparesis. Patients with a past medical history of upper GI surgeries or uncontrolled T2DM were excluded. Risk factors of CKD, including controlled T2DM, essential HTN, and hyperlipidemia, were charted for each group. Specifically, **uncontrolled diabetes** was defined as the presence of ICD-10 codes indicating diabetes with complications, including a history of diabetic ketoacidosis (DKA), hyperosmolar hyperglycemic state (HHS), diabetic retinopathy, retinal detachment, diabetic foot ulcer, or diabetic circulatory complications. **Controlled diabetes** was defined as diabetes without documented complications or diabetes in remission, based on corresponding ICD-10 codes. Patient demographics, including age, race, and gender, were collected. To assess the odds of developing gastroparesis in different stages of CKD, we included patients diagnosed with CKD (**cases**) and compared them to those without CKD (**controls**). All diagnoses included or excluded from this study were identified by the ICD-10-CM code.

For the outpatient database, adult patient records were extracted from the Research USA Minimal Data Shift, by using TriNetX. Inclusion criteria entailed ambulatory visits with records of presenting complaints, including nausea and/or vomiting. Similarly, patients were categorized into two groups: CKD, serving as the case group, and those without CKD, serving as controls. Exclusion criteria for both groups included common risk factors for gastroparesis, including systemic sclerosis, Parkinson’s disease, cystic fibrosis, amyloidosis, other systemic connective tissue diseases, and multiple sclerosis. Patients with a history of gastric or esophageal procedures—including sleeve gastrectomy, Whipple procedure, fundoplication, partial esophagectomy, and catheter ablation for atrial fibrillation—were also excluded. Finally, the use of certain medications, such as anticholinergics, led to exclusion from both groups. A propensity score matching was performed between the two groups based on age, gender, race, medical history of hypertension (HTN), hyperlipidemia, Type 2 Diabetes Mellitus (T2DM), smoking history, and Glucagon-Like Peptide-1 (GLP-1) receptor agonist use. After matching, 404,359 patients from each group were included for comparison.

### 2.3. Statistical Analysis

The study utilized both TriNetX and NIS-sourced data derived from patient demographics and inherent risk factors. To investigate the association between gastroparesis and CKD, chi-squared analysis was performed to compare individuals with CKD to those without. Additionally, multivariate logistic regression analysis was utilized to explore the potential stratified risk of gastroparesis across different stages of CKD through odds ratios with corresponding 95% confidence intervals (CIs). Adjusted odds ratios were calculated while controlling for potential confounders such as age, gender, and race for the inpatient samples. A two-sample test for equal proportions was conducted to assess statistical significance, with a threshold *p*-value of <0.05 considered significant. All statistical analyses were performed using IBM SPSS Statistics, version 28.0.1.1 (IBM Corp., Armonk, NY, USA) for the NIS database analysis, and the embedded analytical tools within the TriNetX platform (TriNetX LLC, Cambridge, MA, USA) for the TriNetX data analysis.

## 3. Results

A total of 7,159,694 hospitalized patients were retrieved and included in this study. Patients with CKD were older than those without CKD, and there were slightly more females than males in each group. The prevalence of controlled T2DM was significantly higher in patients with CKD versus the group without (43.3% vs. 24.9%, *p* < 0.01). A higher proportion of patients with CKD were also diagnosed with hyperlipidemia and hypertension than those without CKD (Table 1).

**Figure 1:**
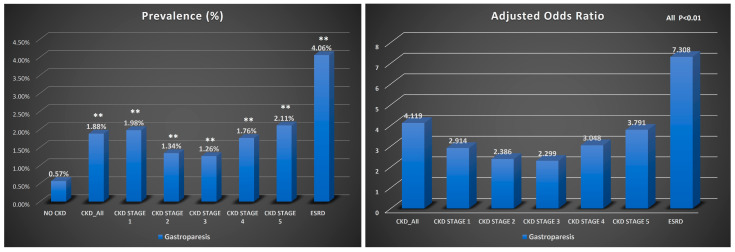
Bar graph showing the prevalence of gastroparesis and adjusted odds ratios in inpatient CKD cohorts, **, *p* < 0.01.

Patients with CKD were more likely to have gastroparesis than those without CKD (OR: 4.287, 95% CI: 4.199–4.377, *p* < 0.001). This increased risk of gastroparesis was observed across different stages of CKD, with a higher predilection towards those with declining renal function and ESRD. The highest risk of gastroparesis was found in patients with ERSD (OR: 8.079 95% CI: 7.874–8.289, *p* < 0.001). The incidence of gastroparesis was 1.88% in patients with CKD, regardless of disease stage, and 0.57% in those without CKD (*p* < 0.001), with an absolute risk difference of 1.31%, also suggesting a higher risk of gastroparesis in the CKD group. This heightened incidence was uniform across all stages of CKD, with the highest incidence of gastroparesis found in patients with ESRD (4.06%, *p* < 0.001) (Table 2 and Figure 1).

**Figure 2:**
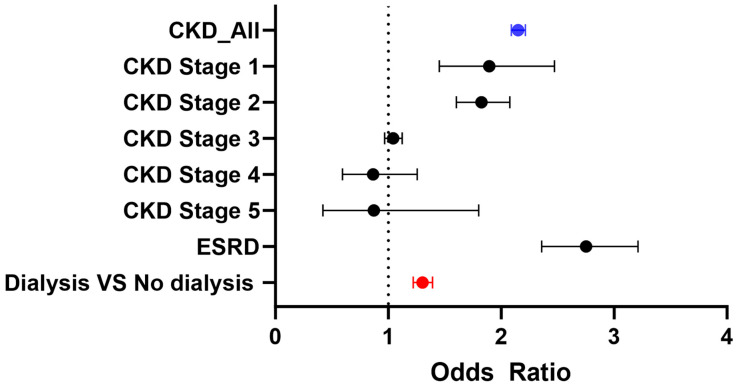
Odds ratios of gastroparesis in outpatient CKD cohorts.

In the outpatient cohort, after applying the inclusion and exclusion criteria, a total of 6,263,251 patients were included before propensity matching. Similar to the inpatient cohort, patients with CKD were generally older and had a higher prevalence of T2DM, hyperlipidemia, and hypertension compared to those without CKD. After propensity matching, 404,359 patients were included in each group (CKD and non-CKD), with an average age of 68 years. Key baseline characteristics—such as T2DM (42%), smoking (8%), hypertension (79%), hyperlipidemia (50.8%), and GLP-1 receptor agonist use (5.8%)—were well balanced between the two groups (Table 1). Consistent with findings in the inpatient cohort, CKD was associated with significantly increased odds of developing gastroparesis, with CKD patients showing more than twice the risk compared to non-CKD patients (OR 2.15, 95% CI: 2.089–2.214). Similar to the inpatient data, the absolute risk difference also indicates a higher risk of gastroparesis in patients with CKD (Table 3, Figure 2). This trend remained significant across CKD stages, including Stage 1 (OR 1.86, 95% CI: 1.452–2.472), Stage 2 (OR 1.83, 95% CI: 1.604–2.077), and ESRD (OR 2.75, 95% CI: 2.358–3.213). Notably, patients receiving dialysis (either hemodialysis or peritoneal dialysis) were at an even higher risk of developing gastroparesis compared to CKD patients not on dialysis (OR 1.30, 95% CI: 1.221–1.392) (Table 3 and Figure 2).

## 4. Discussion

Our most significant outcome in this study is demonstrating an increased risk and incidence of gastroparesis in patients with CKD compared to those without. Further sub-stratification based on stages of CKD revealed that this relationship correlated with advanced CKD; with the risk and incidence of gastroparesis peaking in patients with advanced CKD and ESRD. This is the first study using extensive inpatient and outpatient data to specifically investigate gastroparesis in CKD patients. Our findings suggest that CKD itself may be an independent risk factor for gastroparesis. Even after rigorous propensity score matching to account for shared or confounding risk factors—such as T2DM and the use of GLP-1 receptor agonists—patients with CKD continued to demonstrate a significantly elevated risk of gastroparesis. This association underscores the importance of recognizing and screening for upper GI symptoms in patients with CKD, even when these symptoms are mild or nonspecific.

From a personalized medicine perspective, these results highlight the need for tailored approaches to patient care. Early detection of gastroparesis in CKD patients could enable timely symptom management, reduce the risk of dehydration-induced renal injury, prevent malnutrition, and potentially slow CKD progression. Importantly, our findings extend beyond CKD cases secondary to diabetes or other common etiologies, suggesting that clinicians should maintain a high index of suspicion for gastroparesis across the broader CKD population. This reinforces the value of individualized patient assessment, proactive gastroenterology involvement, and the integration of GI symptom monitoring into CKD management plans.

By identifying CKD as a potential independent risk factor for gastroparesis, this study provides new insight into patient stratification and opens avenues for more personalized, multidisciplinary care strategies aimed at improving outcomes and quality of life in this vulnerable population.

CKD is most commonly caused by diabetes, hypertension, and glomerulonephritis, other than aging [30,31]. It has been reported that diabetes accounts for approximately 34–54% of CKD cases, while hypertension contributes to about 27%. Primary and secondary glomerulonephritis account for roughly 10% of CKD cases, and chronic tubulointerstitial nephritis, renal neoplasms, and cystic kidney diseases together comprise the remaining 10% [32,33]. The mechanism through which patients with CKD develop gastroparesis and gastrointestinal hypomotility is unclear and likely multifactorial. However, the association between CKD and gastroparesis has already been proposed. A recent meta-analysis found that patients with CKD have a higher risk of delayed gastric emptying [5]. This was later echoed in another study that used radio-isotopic examination of 56 hemodialysis patients and found a significant increase in the incidence of gastroparesis in the ESRD population, despite adequate dialysis [6]. Some literature puts forth the dysregulation of insulin homeostasis and the loss of physiological response to insulin levels as potential mechanisms of gastroparesis. Ideally, high insulin levels in hyperglycemic states induce delayed gastric emptying; conversely, lower insulin levels in response to hypoglycemic states induce rapid gastric emptying. Loss of physiologic regulatory function results in impaired gastric motility and symptomatic manifestations of gastroparesis in diabetic patients [12,13,34]. While these findings are often attributed to advanced diabetes related complications, this pattern was consistent across the board in patients with advanced CKD regardless of their concurrent diagnosis of diabetes mellitus [35,36].

Advanced CKD also results in reduced clearance and increased retention of uremic toxins, leading to autonomic dysfunction [37,38] and subsequent impaired gastric motility, delayed gastric emptying, increased small-intestinal transit time, and regional dysregulation of Interdigestive Myoelectric Complex (**IMC**) activity [39,40]. Such changes contributed to the increased risk of Small Intestinal Bacterial Overgrowth (**SIBO**) [7]. Despite structural anomalies and deformities carrying an inherent risk for SIBO, patients diagnosed with SIBO are often found to have no structural deformity that contributes to the increased risk, thereby alluding to impaired functional motility in the more proximal upper gastrointestinal tract as the underlying cause [41,42]. Consequently, this causative relationship sets into motion a self-propagating interdependent cycle between CKD, SIBO, and worsening of gastroparesis through accumulation of bacterial Uremic Retention Molecules (**URM**) [41,43,44,45].

The dysregulation of nNOS and its synthetic function of generating Nitric Oxide (NO) in patients with CKD further contributes to the development of gastroparesis. NO serves as an integral intracellular signaling molecule that is vital in regulating the muscle tone of the Lower Esophageal Sphincter (**LES**). Any impairment in nNOS function can potentially disrupt LES activity, potentially resulting in gastroparesis [46,47]. Numerous animal models with nNOS loss-of-function mutations have indicated similar findings. [48,49,50]. On the other hand, several studies have demonstrated decreased NO production in patients with all stages of CKD through multiple potential mechanisms [51]. Multiple animal studies and clinical studies have indicated an association between CKD and diminished NO levels [52,53]. In a study comprising 13 CKD patients and 9 healthy control individuals, a marked decrease in NO production was observed in the CKD group when compared to the healthy subjects. Additionally, CKD patients exhibited a notable increase in plasma ADMA levels, indicating an elevated presence of asymmetrical dimethylarginine and thereby suggesting suppressed NO production [54]. In a separate study that encompassed 11 patients with ESRD, the total production of NO was found to be reduced by a factor of five in comparison to the control group [55]. This aligns with our discovery that ESRD patients are linked to a greater prevalence and elevated risk of developing gastroparesis compared to individuals in the early stages of CKD. Hence, the heightened prevalence and odds ratio of gastroparesis among CKD patients in our study might be attributed to diminished NO production resulting from their CKD condition.

Our study exhibits several limitations. While the NIS, a nationwide inpatient database, contains comprehensive inpatient data, it does not capture outpatient information, which represents a substantial portion of clinical practice. This limitation was partially mitigated by incorporating data from the TriNetX database. The assessment of gastroparesis in this study was based on ICD-10-CM codes entered across diverse hospital systems and electronic medical records. Although diagnoses were likely made using methods such as imaging, endoscopy, gastric emptying tests, and pathology, these diagnostic approaches were not explicitly documented in either database. Similarly, the identification of risk factors—including smoking, diabetes, medication use (e.g., GLP-1 agonists, TCAs), gastrointestinal procedure history, other medical comorbidities, and alcohol abuse—was also based on ICD-10-CM codes, without establishing a temporal relationship to the diagnosis of gastroparesis. CKD staging was determined solely by ICD-10-CM codes, assuming classification based on estimated glomerular filtration rate (GFR). Furthermore, the number of patients with CKD stage 4 or 5 was limited, potentially underestimating the association between CKD stage and the risk of gastroparesis in both inpatient and outpatient settings. Additionally, the associations we observed were derived from database analyses rather than longitudinal monitoring, and therefore may represent only temporal relationships at specific time points. These findings will require further evaluation in studies with continuous follow-up. Moreover, the possibility of reverse causality between CKD and gastroparesis was not assessed in our study but should be considered in future research. Although gastroparesis is a relatively uncommon condition with limited systemic impact and is unlikely to directly cause CKD, this relationship should nonetheless be examined to confirm the direction of the association.

Further investigation through both retrospective and prospective studies in CKD patients is warranted to confirm this association. As an initial step, a retrospective study involving CKD patients with or without a confirmed diagnosis of gastroparesis at a large single-center institution would help address many of the limitations inherent to database analyses by allowing for more precisely matched comparison groups. This could be followed by a prospective study enrolling patients with ESR who present with gastroparesis symptoms, in which gastric emptying tests are performed to confirm the diagnosis and to evaluate changes in gastroparesis symptoms before and after initiation of renal replacement therapy. Furthermore, a multicenter study involving patients across different stages of CKD, with or without other etiologies of gastroparesis, would further validate these findings and may help establish clinical guidelines for screening CKD patients for gastroparesis.

Our study underscores the clinical importance of actively evaluating CKD patients who present with epigastric symptoms for potential gastroparesis. This association was demonstrated across two independent datasets, even after rigorous propensity matching and statistical adjustment, while stage-specific gradients varied. These findings suggest that gastroparesis may be an underrecognized comorbidity in CKD, particularly in advanced stages, where delayed gastric emptying could further compromise nutritional status, exacerbate symptom burden, and negatively impact overall prognosis. Early recognition and targeted management of gastroparesis should therefore be an integral part of CKD patient care to improve quality of life, minimize complications, and reduce the associated healthcare burden.

## Figures and Tables

**Table 1:** Patient demographics and information.

	Inpatient CKD	Inpatient Non-CKD	Outpatient CKD	Outpatient Non-CKD	Outpatient CKD	Outpatient Non-CKD
			Pre-Propensity matching		Post- Propensity matching	
**Total number of patients**	502,770	3,076,602	422,238	5,841,013	404,359	404,359
**Age**						
	62.4 ± 0.1	47.5 ± 0.1	68.6 ± 17.6 *	37.3 ± 22.8 *	68.0 ± 17.7	68.1 ± 17.6
**Gender**						
Female	234,733 (53.3%)	1,783,893 (63.4%)	229,787(54.5%) *	3,496,771 (60.6%) *	223,309 (55.2%)	222,943 (55.1%)
Male	268,037 (46.7%)	1,292,709 (36.6%)	174,517 (41.4%) *	2,122,644 (36.8%) *	164,201 (40.6%)	164,556 (40.7%)
**Race**						
White	311,646 (53.8%)	1,900,025 (62.4%)	260,541 (61.7%) *	3,545,903 (61.5%) *	252,584 (62.5%)	254,048 (62.8%)
Black	101,463 (27.1%)	423,511 (17.1%)	81,801 (19.4%) *	882,360 (15.3%) *	75,752 (18.7%)	74,776 (18.5%)
Hispanic	47,439 (11.4%)	380,646 (11.3%)	37,771 (9.0%) *	869,601 (15.1%) *	36,787 (9.1%)	37,226 (9.2%)
Asian	13,230 (2.3%)	93,619 (2.2%)	17,020 (4.0%)	232,557 (4.0%)	16,268 (4.0%)	16,037 (4.0%)
**Risk factors**						
T2DM	25,053 (34.3%)	309,459 (24.9%)	186,899 (44.3%) *	353,951 (6.1%) *	169,490 (41.9%)	169,854 (42.0%)
Smoking	49,639 (11.0%)	422,314 (16.1%)	33,950 (8.0%) *	235,692 (4.1%) *	32,301 (8.0%)	32,608 (8.1%)
Hypertension	254,375 (46.6%)	1,001,862 (39.9%)	336,850 (79.8%) *	864,007 (15.0%) *	319,280 (79.0%)	319,253 (79.0%)
Hyperlipidemia	234,552 (52.2%)	640,711 (24.6%)	221,849 (52.6%) *	517,893 (9.0%) *	205,477 (50.8%)	203,178 (50.2%)
GLP-1A	N/A	N/A	24,728 (5.9%) *	67,283 (1.2%) *	23,259 (5.8%)	21,588 (5.3%)

“*” Statistically significant (*p* < 0.05), “**CKD**” Chronic Kidney Disease, “GLP-1” Glucagon-Like Peptide-1 Analogues.

**Table 2:** Inpatient census and risk estimation.

	Gastroparesis				
	Case	Prevalence	*p* Value	Adjusted OR (CI)	*p* Value
**non-CKD**	34,600	0.57%			
**CKD**	18,953	1.88%	<0.01	4.287 (4.119–4.377)	<0.01
**CKD stage 1**	106	1.98%	<0.01	3.541 (2.914–4.302)	<0.01
**CKD stage 2**	655	1.34%	<0.01	2.386 (2.204–2.583)	<0.01
**CKD stage 3**	5035	1.26%	<0.01	2.299 (2.226–2.373)	<0.01
**CKD stage 4**	1926	1.76%	<0.01	3.048 (2.905–3.198)	<0.01
**CKD stage 5**	308	2.11%	<0.01	3.791 (3.381–4.250)	<0.01
**ESRD**	8305	4.06%	<0.01	8.079 (7.874–8.289)	<0.01

**Table 3:** Outpatient census and propensity matching.

		Gastroparesis					
	Census	Propensity Matching	Number of Outcomes	ARD	Odds Ratio	95% CI	*p* Value
CKD	422,238	404,359	14,550	1.80%	2.15	(2.089, 2.214) *	**<0.001**
Non-CKD	5,841,013	404,359	6899				
CKD-Stage 1	5921	5920	159	1.20%	1.895	(1.452, 2.472) *	**<0.001**
Non-CKD	5,656,007	5920	85				
CKD-Stage 2	24,391	24,214	658	1.20%	1.825	(1.604, 2.077) *	**<0.001**
Non-CKD	5,841,013	24,214	365				
CKD-Stage 3	88,479	88,478	1409	0.10%	1.043	(0.967, 1.124)	0.274
Non-CKD	5,841,013	88,478	1352				
CKD-Stage 4	4125	4124	52	−0.20%	0.865	(0.595, 1.257)	0.447
Non-CKD	5,841,013	4124	60				
CKD-Stage 5	629	629	14	−0.30%	0.872	(0.422, 1.802)	0.712
Non-CKD	5,577,009	629	16				
ESRD	12,117	12,116	605	3%	2.753	(2.358, 3.213) *	**<0.001**
Non-CKD	5,466,574	12,116	227				
HD-CKD	30,414	30,413	2192	1.60%	1.304	(1.221, 1.392) *	**<0.001**
HD	387,236	30,413	1710				

“**CKD**” Chronic Kidney Disease, “**HD**” Hemodialysis,, “**ARD**” Absolute Risk Different” “ESRD” End Stage Renal Disease. Statistically significant (*, *p* < 0.05).

## Data Availability

Restrictions apply to the availability of these data. Data were obtained from [TriNetX and NIS] and are available with the permission of [TriNetX and NIS].

## Abbreviations

**Abbreviation**	**Full Term**
CKD	Chronic Kidney Disease
GI	Gastrointestinal
NIS	National Inpatient Sample
HCUP	Healthcare Cost and Utilization Project
TriNetX	TriNetX Research Network
EHR	Electronic Health Record
ICD-10-CM	International Classification of Diseases, 10th Revision, Clinical Modification
T2DM	Type 2 Diabetes Mellitus
HTN	Hypertension
PUD	Peptic Ulcer Disease
FD	Functional Dyspepsia
EE	Eosinophilic Esophagitis
GERD	Gastroesophageal Reflux Disease
URM	Uremic Retention Molecules
BMI	Body Mass Index
ESRD	End-Stage Renal Disease
GLP-1	Glucagon-Like Peptide-1
OR	Odds Ratio
CI	Confidence Interval
SIBO	Small Intestinal Bacterial Overgrowth
nNOS	Neuronal Nitric Oxide Synthase
NO	Nitric Oxide
LES	Lower Esophageal Sphincter
WT	Wild Type
MMC	Migrating Motor Complex
IMC	Interdigestive Myoelectric Complex
ADMA	Asymmetric Dimethylarginine
HD	Hemodialysis
EGD	Esophagogastroduodenoscopy
